# Inverse Design for Robust Inference in Integrated Computational Spectrometry

**DOI:** 10.1002/nap2.70054

**Published:** 2026-03-28

**Authors:** Wenchao Ma, Raphaël Pestourie, Zin Lin, Steven G. Johnson

**Affiliations:** ^1^ Department of Chemistry Massachusetts Institute of Technology Cambridge Massachusetts USA; ^2^ School of Computational Science and Engineering Georgia Institute of Technology Atlanta Georgia USA; ^3^ Bradley Department of Electrical and Computer Engineering Virginia Tech Blacksburg Virginia USA; ^4^ Department of Mathematics Massachusetts Institute of Technology Cambridge Massachusetts USA

**Keywords:** computational spectrometer, integrated optics, on‐chip spectrometer, photonic integrated circuit, topology optimization

## Abstract

We propose an inverse‐design approach for computational spectrometers in which the scattering media are topology‐optimized to achieve better performance in inference of unknown spectra. Unlike traditional end‐to‐end approaches, our inverse design of the scattering media does not need a training set of spectra, a distribution of detector noise, or an inference algorithm. Our approach allows the selection of the inference algorithm to be decoupled from that of the scatterer. For smooth spectra, we additionally devise a regularized reconstruction algorithm based on Chebyshev interpolation, which yields higher accuracy compared with conventional methods in which the spectra are sampled at equally spaced frequencies or wavelengths with equal weights. Our approaches are numerically demonstrated via inverse design of integrated computational spectrometers and reconstruction of example spectra. The inverse‐designed spectrometers exhibit significantly better performance in the presence of noise than their counterparts with random scatterers. Our method provides a useful complement to end‐to‐end co‐design methods.

## Graphical abstract

This work demonstrates that integrated computational spectrometers can be inverse‐designed to substantially improve noise tolerance without relying on training data or specific inference algorithms, offering an alternative to conventional end‐to‐end co‐design strategies.

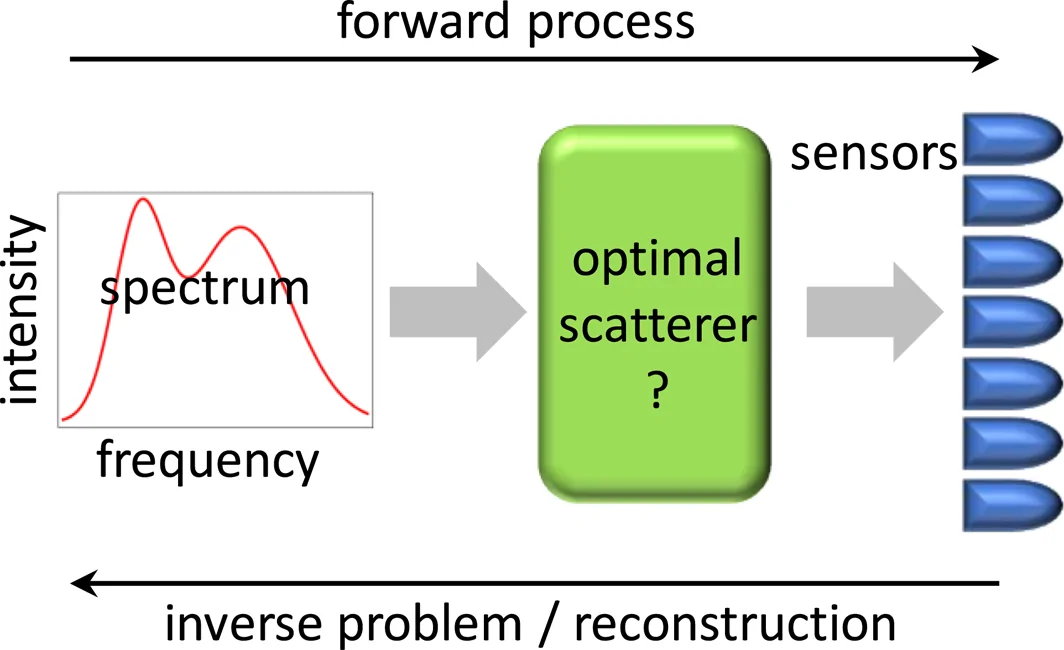

## 1 Introduction

Conventional computational spectrometry (Section 2) attempts to reconstruct the spectrum of input light by analysis of the light scattered through a complex medium (Figure 1), often a disordered medium, exploiting the fact that the recorded signal is a superposition of frequency‐dependent scattering patterns [1, 2, 3]. Although many different algorithms have been applied to this reconstruction [4, 5, 6, 7, 8, 9, 10, 11, 12, 13, 14, 15, 16, 17, 18, 19, 20], usually by some form of optimization/regression problem, previous work typically takes the scattering medium itself as given, or perhaps selects from a small menu of randomized geometries [6]. In this work, we address the question of whether a better scattering medium can be *inverse‐designed* for computational spectrometry, optimizing the medium itself over a vast number $\left(\approx 4\times 10^{5}\right)$ of parameters in order to maximize some measure of “information throughput” and/or robustness against noise for the subsequent computational inference. Of course, given an arbitrarily large scattering volume and enough sensors, one can make computational inference easier simply by designing a prism/demultiplexer (where different wavelengths are designed to scatter to different sensor regions) [8, 21, 22], but the challenge is to obtain accurate reconstruction with a small scatterer (e.g., integrated onto a chip [6, 17, 22, 23, 24, 25, 26, 27, 28, 29, 30]) and a few sensors (e.g., a discrete set of output waveguides).

**FIGURE 1:**
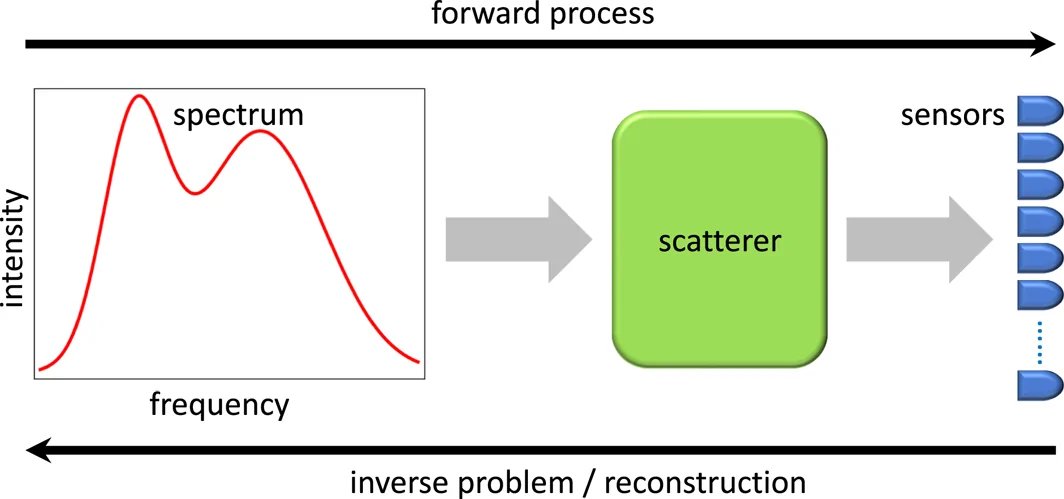
Sketch of computational spectrometry: Forward process and inverse problem. In the forward process, input waves pass through a scatterer and form frequency‐dependent patterns on sensors. If this dependence is calibrated beforehand, one may reconstruct unknown spectra from the signals recorded by the sensors, which is an inverse problem.

We demonstrate that it is possible to achieve order‐of‐magnitude improvement in reconstruction robustness against sensor noise, compared with the median performance of an ensemble of random scatterers, by inverse‐designing the scatterer to improve inference. Rather than “end‐to‐end” design where one co‐optimizes inference and scattering to directly minimize reconstruction error [31, 32, 33, 34, 35]—which requires a training set of spectra and the assumptions on the distribution of detector noise and a reconstruction algorithm as well as various additional hyperparameters such as the mini‐batch size and learning rate in stochastic optimization—we instead show that an efficient and interpretable alternative is to optimize a measure of the “inference robustness” of the scattering system given by a nuclear norm (also called trace norm) [36, 37] of the pseudoinverse of a measurement matrix (relating input spectra to sensor readings). Our nuclear‐norm formulation simultaneously addresses two performance goals: different frequencies should scatter into very distinct sensor readings (leading to “well‐conditioned” reconstruction), but the collection efficiency should also be high at all frequencies (related to improving signal‐to‐noise ratios). We show that our approach is tractable for freeform topology optimization (TopOpt), where “every pixel” is a degree of freedom, demonstrated theoretically in an example two‐dimensional (2d) system modeling an integrated‐optics spectrometer. Our example system employs a single dielectric waveguide as input, passes light with wavelengths λ∈[1540,1560] nm through a scatterer smaller than 10λ, and reconstructs a continuous spectrum using 12 output waveguides. Our optimized structure (which behaves very unlike a prism) exhibits greatly improved robustness against noise for computational inference by least‐squares (overdetermined) reconstruction, even though no explicit noise, training spectra, or reconstruction algorithm were used during the scatterer optimization itself. The fact that our objective function is directly related to the singular values of the measurement matrix allows us to immediately interpret why this robustness occurs. Another key challenge is relating discrete measurements to reconstructing a spectrum over a continuous frequency range. For spectra that vary smoothly with frequency, we devise a regularized reconstruction algorithm that exploits this smoothness using Gauss–Legendre quadrature [38] and Chebyshev polynomial interpolation [39, 40], yielding greater accuracy than typical methods in which the spectrum is sampled at equally spaced and equally weighted points. We believe that our approach, which separates the design of an improved scatterer from the specifics of reconstruction, should enable efficient exploration of future computational spectrometry systems and algorithms (Section 5), and related approaches may also be applicable to designing optics for computational imaging [41, 42, 43, 44, 45, 46] or other inference problems [34].

Inverse design employs large‐scale optimization to maximize optical performance, measured by some figure of merit (FOM), over a huge number of geometric and/or material degrees of freedom (DOFs) [47, 48]. Specifically, in this work we employ topology optimization (TopOpt) [49, 50, 51], in which freeform geometries are optimized over “every pixel” of the structure, typically while imposing manufacturing constraints [51, 52]. A key enabling factor of inverse design is that one can compute the derivatives (i.e., the gradient) of an FOM with respect to a huge number of DOFs (e.g., the material at every pixel) by a single additional “adjoint” solve of essentially the same (Maxwell) equations [47, 48, 53], which can then be used for gradient ascent and related optimization algorithms [54, 55, 56]. When the output of the optics is fed into computational inference, however, one should target a different FOM: instead of designing optics for a pre‐determined wave‐scattering operation (e.g., a coupler), one wishes to minimize the error of the inference in the presence of noise. The most direct approach to improving inference is end‐to‐end co‐design: given a training set of inputs and a distribution of detector noise, one minimizes the mean error in the final inference with respect to geometry and inference DOFs (backpropagating the gradient of the error through both the inference and the Maxwell solves), and this strategy has been recently applied to several problems in imaging and other applications [31, 32, 33, 34, 35]. The end‐to‐end approach requires a training set, a noise distribution, and an inference model. Incorporating explicit noise and/or random training‐set sampling leads towards stochastic‐optimization algorithms [57], excluding many other optimization methods unless additional approximations are made. Some authors instead optimize a deterministic training‐data–free proxy for inference robustness, such as measures of mutual information [58] or Fisher information [59], which are related to our nuclear‐norm formulation (Section 3.2). We describe an explicit comparison to end‐to‐end methods for our spectrometry example in Section 4.3. Related work has instead sought theoretical upper bounds on the information throughput of optical systems, for example, in terms of channel capacity [60, 61, 62, 63], although these bounds do not identify specific practical designs.

## 2 Computational Spectrometer

### 2.1 Forward Model

In a conventional computational spectrometer, light passes through a scatterer and forms frequency‐dependent patterns on sensors (One can also employ multiple measurements through a reconfigurable scatterer on a single sensor [17, 26, 27, 28]). Output signals typically depend linearly on input power and the incoming light is incoherent at different frequencies. For a spectrometer with a finite number of sensors as sketched in Figure 1, the power received by each sensor is an integrated power over a range of frequencies. In the presence of noise, the signal recorded at the kth sensor can be written as follows:

(1) $$v_{k}=\int F_{k}\left(\omega \right)u\left(\omega \right)d\omega +\zeta_{k},$$

where ω denotes frequency, u(ω) denotes the input power at the frequency ω (i.e., the unknown spectrum to be determined below), $F_{k}\left(\omega \right)$ denotes the signal recorded at the kth sensor due to unit input power at the frequency ω, $\zeta_{k}$ denotes the noise on the kth sensor, and $v_{k}$ denotes the power received by the kth sensor. The function $F_{k}\left(\omega \right)$ describes the overall response the optical system between input and output ends, encapsulating the effects of various components, such as sensors, scattering media, filters, and substrates.

For a given spectrometer, estimating u(ω) (the spectrum) from all $v_{k}$ (the sensor measurements) and $F_{k}\left(\omega \right)$ (which can be determined from simulation or experimental calibration) requires decomposing u(ω) into a finite number of unknowns, such as expanding u(ω) in terms of a basis of functions with unknown coefficients, or discretizing frequency ω into a finite number of samples $\omega_{j}$ at which each $u\left(\omega_{j}\right)$ is unknown. Solving for these unknowns results in spectral reconstruction.

In a discretization scheme, the exact integral can be approximated by a weighted sum with weights $w_{j}$ at a set of discrete frequencies $\omega_{j}$ (according to a quadrature rule [64] and the domain of integration):

(2) $$v_{k}\approx \sum_{j}w_{j}F_{k}\left(\omega_{j}\right)u\left(\omega_{j}\right)+\zeta_{k}\;⟺\;v\approx FWu+\zeta \;,$$

where on the right we have expressed the relation in matrix form: u, v, and ζ are column vectors of $u\left(\omega_{j}\right)$, $v_{k}$, and $\zeta_{k}$; $W=diag\left(w_{1},w_{2},\text{⋯}\;\right)$ contains the weights. For example, the simplest quadrature rule is a Riemann sum with equally spaced frequencies $\omega_{j}=\omega_{0}+j\Delta \omega$ and equal weights $w_{j}=\Delta \omega$ [64].

One may also expand $u\left(\omega \right)\approx \sum_{\ell }b_{\ell }\left(\omega \right)c_{\ell }$ in some finite set of basis functions $b_{\ell }$ (e.g., polynomials such as the Chebyshev polynomials [39], radial basis functions [65] such as Gaussians [18, 19, 20, 66], etc.) and unknown coefficients $c_{\ell }$; in terms of this basis, the matrix equation and the spectrum vector in Equation (2) can be expressed as follows:

(3) v≈FWBc+ζ,

where B is a matrix with elements $B_{j,\ell }=b_{\ell }\left(\omega_{j}\right)$ and c is a column vector of $c_{\ell }$. If B is an identity matrix, c becomes u and the matrix equation in Equation (3) becomes identical to that in Equation (2).

### 2.2 Reconstruction

The key enabling factor for reconstruction is that the columns of F are distinct: different frequencies yield different measured signals, allowing one to disentangle superpositions of multiple frequencies into their component amplitudes. In this work, we consider only overdetermined problems, in which more measurements than unknowns are available. In terms of Equations (2) and (3), the vector v contains more elements than u or c.

To reconstruct an unknown continuous spectrum, a conventional approach is to seek a least‐squares solution, corresponding to using the pseudoinverse of the matrix FW in Equation (2):

(4) $$\hat{u}={\left(FW\right)}^{+}v,$$

where $\hat{u}$ denotes the reconstructed spectrum and the superscript + denotes pseudoinversion [67], which becomes ordinary matrix inversion if FW is a square matrix. The continuous spectrum can then be recovered via interpolation and extrapolation (or only interpolation if the discrete frequencies span the full range of interest). One may also estimate the coefficients of the basis function in Equation (3) and then reconstruct the spectrum:

(5) $$\hat{c}={\left(FWB\right)}^{+}v,\;\hat{u}=B\hat{c},$$

without the need for an extra interpolation or extrapolation step. Although the spectrometry problem is underdetermined because intensities at infinitely many frequencies are reconstructed from a finite number of measurements, the basis functions impose a smoothness prior that allows the problem to become determined or overdetermined if the number of reconstructed coefficients does not exceed the number of measurements.

To reduce reconstruction error further for overdetermined problems in the presence of noise, Tikhonov regularization [68] is typically employed. In terms of expansion with basis functions in Equations (3) and (5), one can estimate the coefficients and spectra as follows:

(6) $$\hat{c}=\arg\min_{c}\left({\left\|FWBc-v\right\|}_{2}^{2}+\alpha {\left\|\sqrt{W}Bc\right\|}_{2}^{2}\right),$$

where α is a regularization coefficient. The estimated coefficients $\hat{c}$ can be analytically solved and an unknown spectrum can then be reconstructed:

(7) $$\begin{array}{ccc} \hat{c} & = & {\left[B^{⊤}\left(WF^{⊤}FW+\alpha W\right)B\right]}^{-1}{\left(FWB\right)}^{⊤}v,\\ \hat{u} & = & B\hat{c}. \end{array}$$

Other computational spectrometry work, especially in the underdetermined case, has also explored other methods such as sparsifying $L_{1}$ regularization [13, 14, 15, 16, 17, 28, 69, 70] and even neural networks [71, 72, 73, 74, 75, 76].

## 3 Inverse‐Design Method

### 3.1 Performance Metrics

In this work, we focus on two performance metrics: robustness against noise on sensors and collection efficiency of signals (which is related to signal‐to‐noise ratios).

The robustness is related to the ratio of the relative error in the reconstructed spectrum, namely $\left\|\hat{u}-u\|/\|u\right\|$, to the relative error in the signal, namely ‖ζ‖/‖v‖. This ratio is bounded above by the condition number of the matrix $F\sqrt{W}$ [67], up to the discretization error in ω. To see how robustness connects with $F\sqrt{W}$, we first consider the difference between the true and reconstructed spectra when Equation (2) is used:

(8) $$\hat{u}-u\approx {\left(FW\right)}^{+}\zeta =W^{-1}F^{+}\zeta ,$$

where the last equality relies on the assumption that F has linearly independent columns, which requires that frequencies are no more than measurements. We quantify the error of reconstruction as follows:

(9) $$\begin{array}{cc} & \sqrt{\int {\left[\hat{u}\left(\omega \right)-u\left(\omega \right)\right]}^{2}d\omega }\approx \sqrt{\sum_{i}{\left[\hat{u}\left(\omega_{i}\right)-u\left(\omega_{i}\right)\right]}^{2}w_{i}}\\ & =\sqrt{{\left(\hat{u}-u\right)}^{⊤}W\left(\hat{u}-u\right)}=\sqrt{{\left(F^{+}\zeta \right)}^{⊤}W^{-1}\left(F^{+}\zeta \right)}\\ & ={\left\|{\left(F\sqrt{W}\right)}^{+}\zeta \right\|}_{2}, \end{array}$$

with ⊤ denoting matrix transposition, and $\|\cdot {\|}_{2}$ denoting the $L_{2}$ norm. Likewise, we have the following equation:

(10) $$\sqrt{\int u{\left(\omega \right)}^{2}d\omega }\approx \sqrt{\sum_{i}u{\left(\omega_{i}\right)}^{2}w_{i}}={\left\|\sqrt{W}u\right\|}_{2}.$$

Therefore, the ratio of the relative error in the reconstructed spectrum to the relative error in the signal is as follows:

(11) $$\begin{array}{cc} & \frac{\sqrt{\int {\left[\hat{u}\left(\omega \right)-u\left(\omega \right)\right]}^{2}d\omega }}{\sqrt{\int u{\left(\omega \right)}^{2}d\omega }}/\frac{\|\zeta {\|}_{2}}{\|FWu{\|}_{2}}\\ & \approx \frac{{\left\|{\left(F\sqrt{W}\right)}^{+}\zeta \right\|}_{2}}{{\left\|\sqrt{W}u\right\|}_{2}}/\frac{\|\zeta {\|}_{2}}{\|FWu{\|}_{2}}\\ & =\frac{{\left\|{\left(F\sqrt{W}\right)}^{+}\zeta \right\|}_{2}}{\|\zeta {\|}_{2}}\frac{\|F\sqrt{W}\sqrt{W}u{\|}_{2}}{{\left\|\sqrt{W}u\right\|}_{2}}. \end{array}$$

As a standard approach is then to maximize over ζ and u [67], in which case the two factors after the equality become the $L_{2}$‐induced matrix norms $\|{\left(F\sqrt{W}\right)}^{+}{\|}_{2}$ and $\|F\sqrt{W}{\|}_{2}$, the product of which is the condition number of the matrix $F\sqrt{W}$ defined with the same norm, which is also the ratio between the maximum and minimum singular values [67]:

(12) $$\kappa \left(F\sqrt{W}\right)={\left\|F\sqrt{W}\right\|}_{2}{\left\|{\left(F\sqrt{W}\right)}^{+}\right\|}_{2}=\frac{\sigma_{\max}\left(F\sqrt{W}\right)}{\sigma_{\min}\left(F\sqrt{W}\right)}.$$

As an upper bound of the ratio of relative errors in Equation (11), the condition number can be regarded as a performance metric of a computational spectrometer.

A low condition number, however, is not sufficient for good performance: if all of the singular values are small, that would signify low collection efficiency, even if the σ ratios are close to 1. Large signal intensities are beneficial for robust inference in the presence of noise components that do not increase (e.g., background light) or increase sublinearly with signal intensities (e.g., shot noise, which increases as $∼\sqrt{\text{intensity}}$ [77]). Therefore, the collection efficiency, for example, transmittance in a transmission spectrometer, should also be a performance metric. Here we report a straightforward way to incorporate both criteria (high collection efficiency and low condition number) into a single differentiable figure of merit, described in Section 3.2 below.

This efficiency can be considered as the ratio of the power of signals recorded by sensors (in the limit of no noise) to the power of an input spectrum with uniform intensity across the operational frequency range:

(13) $$\eta =\int \sum_{k}F_{k}\left(\omega \right)\approx \sum_{j,k}w_{j}F_{k}\left(\omega_{j}\right)\;.$$

### 3.2 Objective Function

A spectrometer with good performance should be robust against noise on sensors while having acceptable collection efficiency. From Equations (11) and (12) the condition number κ≥1 should be low, whereas the efficiency η from Equation (13) should be high. To simultaneously account for both performance metrics in gradient‐based optimization, instead of formulating a multi‐objective optimization problem, we introduce a single figure of merit (FOM) to be *minimized*:

(14) $${\left\|{\left(F\sqrt{W}\right)}^{+}\right\|}_{*}=tr\;\left[{\left(WF^{⊤}F\right)}^{-1/2}\right]=\sum_{j}\frac{1}{\sigma_{j}}.$$

where $\|\cdot {\|}_{*}$ means taking the nuclear norm (also called trace norm) [36, 37] and $\sigma_{j}$ denotes each singular value of $F\sqrt{W}$. (Using the trace expression here facilitates the computation of gradients and makes this objective compatible with automatic differentiation. The flow chart is illustrated in Figure 2a.) Clearly, minimizing this FOM tries to make all the singular values $\sigma_{j}$ larger, which implies higher collection efficiency for all relevant input spectra. On the other hand, the collection efficiency is bounded above by 100%, indicating the existence of upper bounds on these singular values. Therefore, making them larger tends to decrease the *spread* of singular values. In particular, the FOM is dominated by and has the sharpest dependence on the smallest $\sigma_{j}$, implying that this singular value is likely to enjoy the most relative increase as the FOM is minimized. Consequently, both a low condition number and a high collection efficiency are encouraged. We observe these two effects below in Figure 3.

**FIGURE 2:**
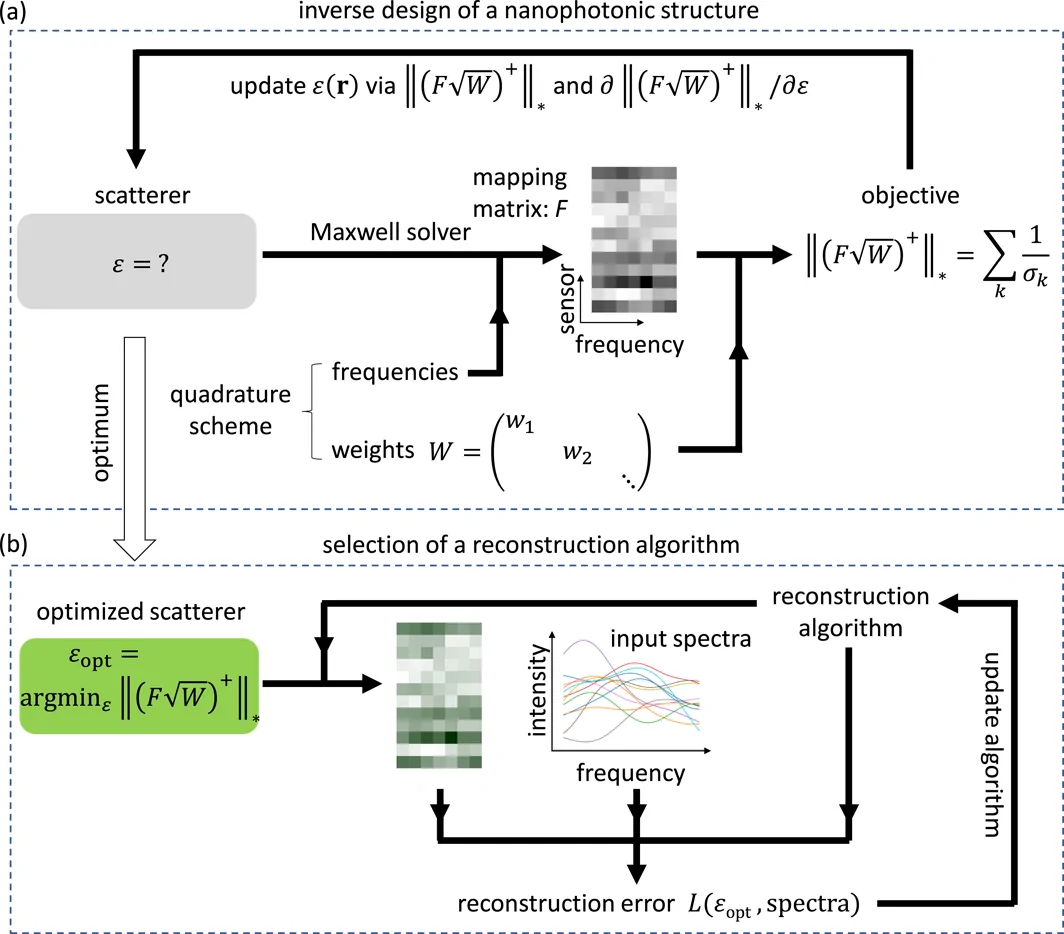
Framework of design methods. (a) Inverse design of a nanophotonic structure. The frequencies at which the spectral–spatial mapping matrix (also called the measurement matrix in this paper) is computed are determined by the frequency range and the quadrature scheme. This mapping matrix and the quadrature weights determine the objective function. (b) Selection of a reconstruction algorithm. After the optimized scatterer is obtained, given prior knowledge of input spectra, one can select a reconstruction algorithm based on reconstruction error.

**FIGURE 3:**
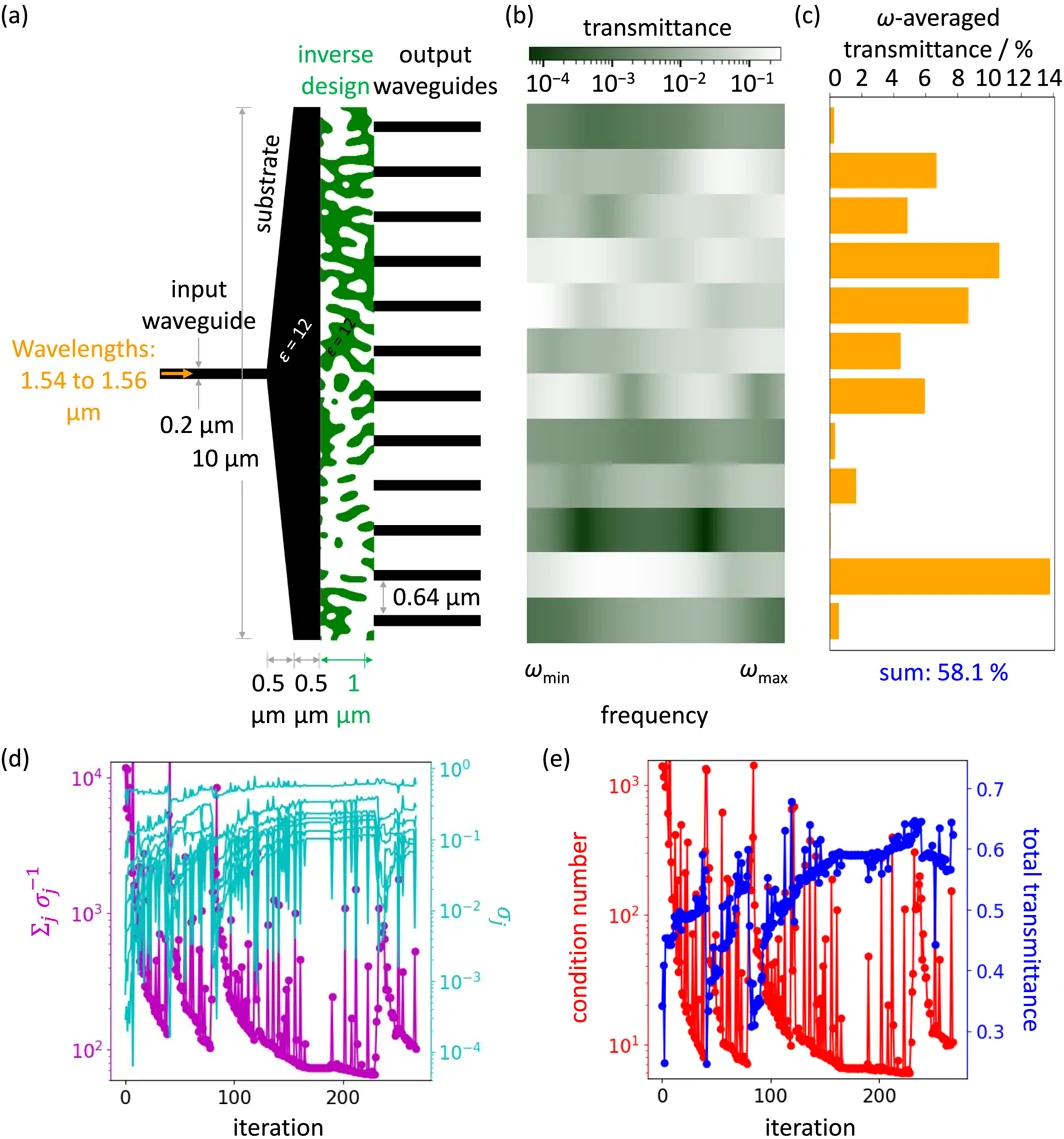
Inverse design of an integrated spectrometer. (a) Structure of the spectrometer. This 2d device consists of an input waveguide, a wedge structure, a design region, and 12 output waveguides, with the solid material having a relative permittivity ≈12. The width of all the waveguides is 0.2 μm. Adjacent output waveguides are separated by 0.64 μm. (b) Transmittance of the optimized spectrometer at each output waveguide across the frequency range of interest. (c) Frequency‐averaged transmittance of the optimized spectrometer at each output waveguide. The total transmittance is 58.1%. (d) Objective function $\left(\sum_{j}\sigma_{j}^{-1}\right)$ and singular values of $F\sqrt{W}$ during optimization. (e) Condition number and total transmittance during optimization, computed from the 12×7 spectral–spatial mapping matrix.

In our approach, the optimization can evaluate transmission at relatively few frequencies (few columns of F), corresponding to a low‐order quadrature scheme. In contrast, modeling the reconstruction step requires one to explicitly compute the measured noisy‐sensor readings v accurately for training/test spectra, involving a denser set of frequencies. An end‐to‐end approach which directly incorporates reconstruction into optimization can thus be more computationally costly. Furthermore, the use of a deterministic objective function in our approach allows a wide range of optimization algorithms to be employed, including algorithms that support nonlinear constraints, such as the manufacturing constraints that are almost always required in practical TopOpt [78, 79]. Explicit random noise and sampling of training data in end‐to‐end methods implies a smaller menu of stochastic‐optimization methods [57], which have more limited options for nonlinear constraints [80].

Many other objective functions may have similar effects, such as $tr\left[{\left(WF^{⊤}F\right)}^{-1}\right]=\sum_{j}{\sigma_{j}}^{-2}$ which is implied by the Fisher information [81], and $-\ln\;\det\left(WF^{⊤}F\right)=-2\sum_{j}\;\ln\sigma_{j}$ [82]. In our test example discussed in Section 4, the former objective function yields an optimized structure with performance comparable to that of the design from Equation (14), whereas the log‐determinant objective leads to much worse performance. Further variations of these objective functions could be explored, such as $tr\left\{{\left[{\left(WF^{⊤}F\right)}^{-1}\right]}^{n/2}\right\}=\sum_{j}{\sigma_{j}}^{-n}$ with n>0 (apart from n=1 or 2 that result in the objective functions mentioned earlier). One may also separately design a target matrix for F (which may not be physically attainable) and then optimize the structure to minimize the distance of its actual F to this target [83] but this approach may over‐constrain the design by not allowing it the freedom to discover the best attainable F.

In this work, we choose the discrete frequencies $\omega_{j}$ and weights $w_{j}$ according to a Gauss—Legendre quadrature rule, in order to maximize integration accuracy for a given number of points [38]. This determines the matrix W. Below, we used a 7‐point quadrature rule, appropriate for the smooth example spectra considered in our tests, but in general this choice will depend on the system of interest. Enlarging the number of quadrature points increases the computational cost, although this is somewhat ameliorated by our use of a hybrid time/frequency‐domain scheme that computes all frequencies simultaneously [84].

### 3.3 Topology Optimization

For inverse design, we adopt density‐based topology optimization, in which the design region is meshed and a “density” related to the permittivity at each pixel is a parameter to be optimized [49]. This density ρ(x) is defined on the design region and ranges in [0, 1]. Before computing the permittivity, the density is blurred and then projected. The blurring operation can be described as convolution with a filter: $\tilde{\rho }\left(x\right)=w*\rho$, where $\tilde{\rho }$ is the density after blurring, and we choose the filter w as a conic filter, the radius of which is related to the minimum lengthscale of the design pattern [52]. After blurring, a projection operation is performed to compute an almost everywhere binary density $\hat{\tilde{\rho }}$, with a hyperparameter β representing the binarization strength [85]. The permittivity in the design region is then

(15) $$\epsilon =\epsilon_{\min}+\left(\epsilon_{\max}-\epsilon_{\min}\right)\hat{\tilde{\rho }},$$

where $\epsilon_{\min}$ and $\epsilon_{\max}$ are the minimum and maximum permittivities in the design region. In optimization, one usually starts with small β and gradually increases it, so that the structure becomes binarized. In this work, we used β= 2, 4, 8, 16, 32, and ∞, each of which spanned a number of iterations. After these iterations, minimum lengthscale constraints were imposed along with β=∞ to prevent too small geometric features in the final design [86].

During conventional density‐based topology optimization, the structural parameters are updated by gradient‐based optimization algorithms, in particular the CSSA algorithm (conservative convex separable approximation) with either the method of moving asymptotes (CCSA‐MMA) or a quadratic penalty (CCSA‐Q) [87]. In this work, we adopted CSSA‐MMA before imposing lengthscale constraints and then CCSA‐Q during the final set of iterations with lengthscale constraints. Both algorithms are implemented in NLopt, a free and open‐source software package [88]. The gradient of the objective (the trace expression in Equation (14)) with respect to structural parameters can be rapidly obtained from an adjoint method, which consists of two simulations: the forward simulation of the original problem, and the adjoint simulation in which the adjoint sources related to the output instead of the input are placed. All electromagnetic simulations in optimization and verification were performed with a free and open‐source implementation of the finite‐difference time‐domain (FDTD) method [89] and the inverse design was performed with its hybrid time/frequency‐domain adjoint module [84].

## 4 Results and Discussions

### 4.1 Example Structure

Here, we demonstrate our methods on a simple two‐dimensional (2d, xy) example of an integrated spectrometer. As Figure 3a shows, the structure consists of an input waveguide, a wedge region, a design region, and 12 output waveguides, where the solid material has a relative permittivity of $3.48^{2}$ (≈12, like silicon). (An alternative example system, in which the output waveguides are replaced by a uniform medium and far‐field sensors, is given in Section S5 in Supporting Information S1.) All waveguides have a width of 0.2 μm and the separation between output waveguides is 0.64 μm. Incoming waves at wavelengths 1.54–1.56 μm with out‐of‐plane $\left(E_{z}\right)$ polarization enter the wedge region from the input waveguide and undergo multiple scattering in the design region, the size of which is 10 μm × 1 μm. The scattering process, which is frequency‐dependent, results in different output patterns at different frequencies, as Figure 3b shows. The input/output waveguides are single‐mode in this wavelength range. We selected seven frequencies according to Gauss–Legendre quadrature of $\int_{1.54}^{1.56}d\lambda$ for performing inverse design, so the size of the spectral–spatial mapping matrix is 12×7.

### 4.2 Inverse Design

During minimization of Equation (14), the smallest singular value increases most significantly, as Figure 3d shows. Meanwhile, as Figure 3e shows, the condition number of $F\sqrt{W}$ decreases from >1000 to <100, and the collection efficiency, which is the transmittance for this structure, increases from 30∼40% to approximately 60%. This high transmittance is unevenly distributed across the output waveguides, 7 of which dominate signal collection while 5 of which collect low portions of light, as shown in Figure 3b,c. This behavior can be explained by the choice of 7 frequencies for optimization, which does not need to make full use of the 12 output channels. As the green–white region in Figure 3a illustrates, in the optimized design, a few high‐transmittance output waveguides are not even connected to the high‐index medium (silicon). This feature, although possible in 2d, would probably be absent in 3d in the presence of out‐of‐plane scattering (Note that the convergence history in Figure 3d is non‐monotonic because we are also showing the “inner” iterations of the CCSA algorithm, in which it aggressively takes too large a step and backtracks by increasing a penalty [87]; there is also a discontinuity each time β is increased.).

The optimized design pattern has a minimum lengthscale of 80 nm, measured by a free and open‐source tool based on morphological transformations [51]. In comparison with some randomly generated structures with the same minimum lengthscale, the optimized structure clearly has better performance in its combination of low condition number and high transmittance, as Figure 4 shows. The performance metrics were evaluated using the 7 frequencies of the Gauss–Legendre nodes. To estimate the influence of fabrication imperfections [90, 91], we also simulated the morphological dilation and erosion by 10 nm of the structures, as also illustrated in Figure 4: The condition number worsens by a factor of 2–3, and is still far better than typical random structures. It is also known that a low condition number makes the resulting inference error robust to perturbations in both the signal vector v (e.g., measurement noise) and the matrix F (e.g., manufacturing error) [67]. (Additional simulations of fabrication imperfections are shown in Section S2 in Supporting Information S1.) As depicted in the right panel of Figure 4, the order‐of‐magnitude smaller condition number corresponds to more‐distinct columns in the 12×7 spectral–spatial mapping matrix of the optimized design. As we show explicitly below, this translates to greater robustness to noise in the reconstructed spectrum.

**FIGURE 4:**
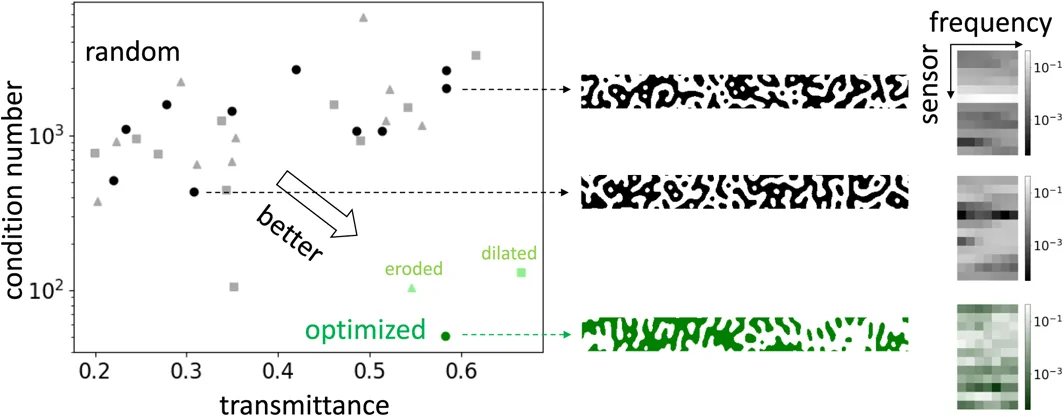
Comparison of performances of random and optimized structures. The horizontal and vertical axes represent the total transmittance and the condition number, respectively. The bottom right area in this coordinate system is associated with lower condition numbers, higher collection efficiencies, and hence better performance. The round green and black dots correspond to the optimized and random structures with the same minimum lengthscale, while the square/triangular symbols with light colors correspond to structures in which solid regions are dilated/eroded by 10 nm. The optimized structure and two random structures are illustrated on the middle panel. Their corresponding 12×7 spectral–spatial mapping matrices are on the right panel.

### 4.3 Reconstruction and Impact of Noise

Once the permittivity pattern is optimized, the first phase of design, as sketched in Figure 2b, is finished. In this section, we turn to the second phase as sketched in Figure 2c, in which reconstruction algorithms are evaluated. Our sample spectra were randomly generated according to the following equation:

(16) $$\langle u\left(\omega_{1}\right)u\left(\omega_{2}\right)\rangle =\exp\left[-\frac{{\left(\omega_{1}-\omega_{2}\right)}^{2}}{\omega_{\text{corr}}^{2}}\right],\;\;\;\;\;\;\langle u\left(\omega \right)\rangle =u_{\text{avg}},$$

where the correlation $\omega_{\text{corr}}$ is chosen as 40% of the frequency range and the average intensity $u_{\text{avg}}$ is chosen as 3. Such spectra have smooth profiles and are usually positive everywhere. Four examples are shown as black curves in Figure 5. We assume independently and identically distributed (i.i.d.) noise on each sensor, obeying a normal distribution with zero mean and standard deviation proportional to the signal intensity on that sensor:

(17) $$\zeta_{k}∼N\left(\mu ,\sigma_{k}^{2}\right),\;\;\;\;\;\;\mu =0,\;\;\;\;\;\;\sigma_{k}=qv_{k},$$

where q describes the relative level of noise. To emulate the forward process, one needs to compute the signals faithfully. Here, we applied Equation (2) using Gauss–Legendre quadrature with 101 points. These densely packed quadrature nodes in the frequency range of interest allow the signals to be computed accurately. On the other hand, the frequencies used for reconstruction hinge on the choices of reconstruction algorithms and may not be the same as those in computing the forward process.

**FIGURE 5:**
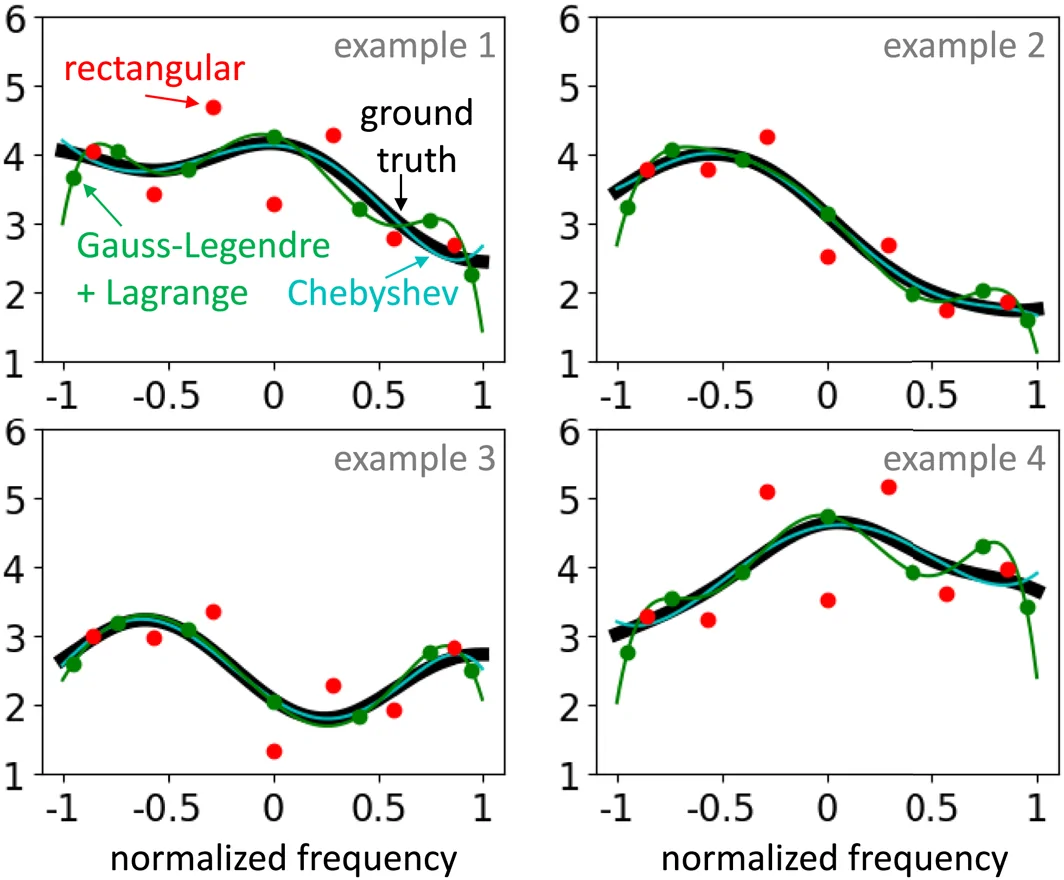
Spectral reconstruction with four examples. The ground truth is plotted as thick black curves. The red dots represent reconstructed spectra at 7 equally spaced frequencies, chosen according the rectangular rule. The green dots represent reconstructed spectra at 7 frequencies chosen according to Gauss–Legendre nodes, while their Lagrange interpolating polynomials are plotted as green curves. The cyan curves represent reconstructed spectra as a linear combination of the first 7 Chebyshev polynomials of the first kind.

As a natural choice, as described by Equation (4), one may reconstruct unknown spectra at a discrete set of frequencies, in particular, equally spaced frequencies. To avoid an underdetermined inverse problem, the number of such discrete frequencies should not exceed the number of sensors, which is 12 in our case. For example, let us consider 7 equally spaced frequencies chosen as the midpoints of intervals in the rectangular rule. Even in the absence of sensor noise ζ, the reconstructed spectral intensities at these frequencies deviate from the true spectra, as shown by the red dots in Figure 5, due to discretization error. Reconstruction at Gauss–Legendre nodes suffers from smaller error, as depicted by the green dots. One can then perform Lagrange interpolation and extrapolation to reconstruct continuous spectra, represented by the green curves.

Alternatively, one may also reconstruct unknown spectra as a linear combination of basis functions, as described by Equation (5). To avoid an underdetermined inverse problem, the number of basis functions should not exceed the number of sensors, while the number of frequencies is unlimited. For example, let us use Chebyshev polynomials of the first kind as the basis functions [39, 40]. Accordingly, B in Equation (5) is a Chebyshev–Vandermonde matrix with rows and columns corresponding to different frequencies and different Chebyshev polynomials, respectively. To make discretization error negligible, we used 101 frequencies located at the Gauss–Legendre quadrature nodes, which implies that W is a 101×101 matrix. With 7 (0th‐ to 6th‐order) such polynomials and the same densely packed frequencies as the forward model, in the absence of sensor noise ζ, the reconstructed spectra, shown as the cyan curves in Figure 5, closely match the ground truth.

Reconstruction errors generally increase with the noise level, as illustrated in Figure 6a. Here, the simulation is based on 2000 randomly generated spectra satisfying Equation (16), each of which suffers from sensor noises described by Equation (17), and the noise in each spectrum is independent. We consider reconstruction from both 6 and 7 basis functions or interpolation points: changing the number of degrees of freedom, leading to well‐known trade‐off between accuracy and robustness. Although 6‐point reconstruction at Gauss–Legendre nodes does not exhibit advantages, reconstruction from 6 (0th‐ to 5th‐order) Chebyshev polynomials appears to be more robust against sensor noise. Tikhonov regularization [68] can decrease reconstruction error further. As Figure 6b shows, for schemes with 7 and 6 Chebyshev polynomials, at the relative noise level q=0.01, with a properly chosen regularization coefficient α, the median reconstruction errors decrease by 55.9% and 6.4%, respectively, from those without regularization. The simulation here is based on $10^{5}$ randomly generated spectra. We also explored reconstruction with Gaussian basis functions (a form of radial basis function [65]) similar to Refs. [20, 66] and found that they could obtain accuracy similar to the Chebyshev polynomials, but required careful tuning of the widths of Gaussians (see Section S1 in Supporting Information S1).

**FIGURE 6:**
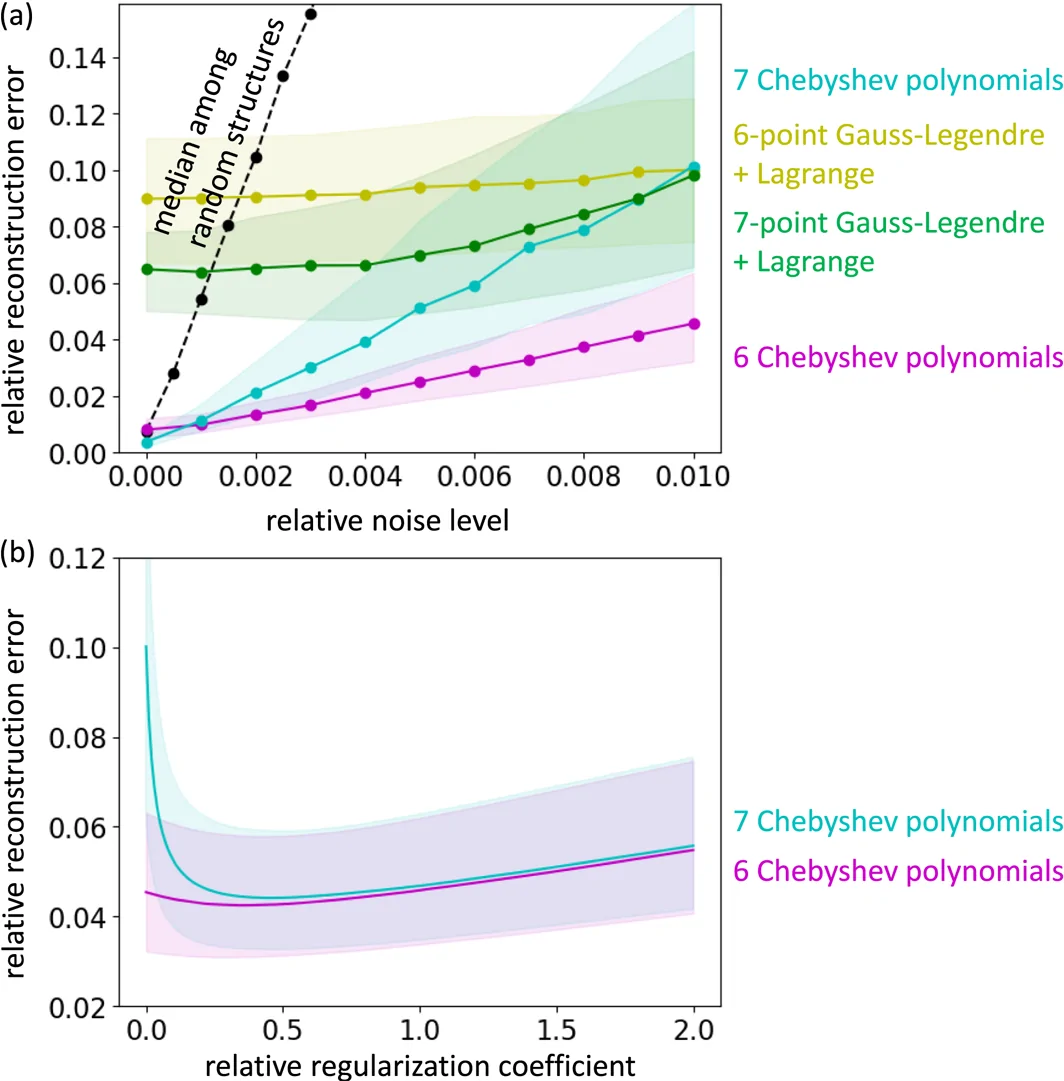
(a) Dependence of reconstruction error on relative noise level. The horizontal and vertical coordinates represent the relative level q of sensor noise in Equation (17) and relative reconstruction error $\sqrt{\frac{1}{\omega_{\max}-\omega_{\min}}\int {\left[\hat{u}\left(\omega \right)-u\left(\omega \right)\right]}^{2}d\omega }/\langle u\left(\omega \right)\rangle$ with $\langle u\left(\omega \right)\rangle =u_{avg}$, where the integral is evaluated at 101 frequencies located at the (scaled) Gauss–Legendre quadrature nodes. Each dot represents the median among 2000 cases with randomly generated spectra and noise according to Equations (16) and (17). Each error band ranges from the first to third quartiles. The dots on the solid and dashed curves are based on the optimized and random structures, respectively. Here, the reconstruction errors for random structures are only for the reconstruction with 6 Chebyshev polynomials. The random structures, corresponding to the circular dots in Figure 4, have the same minimum lengthscale as the optimized structure. (b) Reconstruction errors with Tikhonov regularization. The horizontal coordinate represents a relative regularization coefficient, which we defined as $\alpha /{\langle \sigma \rangle }_{\text{geo}}$ with ${\langle \sigma \rangle }_{\text{geo}}$ denoting the geometric mean of the singular values of $F\sqrt{W}$ evaluated at the 7 Gauss—Legendre quadrature nodes (scaled to the frequency range). Each curve represents the median among $10^{5}$ cases with randomly generated spectra and noise. For the schemes with 7 and 6 Chebyshev polynomials at a relative noise level q=0.01, the optimal regularization coefficients α, at which the median reconstruction errors attain their minima, are $0.85{\langle \sigma \rangle }_{\text{geo}}$ and $0.71{\langle \sigma \rangle }_{\text{geo}}$, respectively. Correspondingly, the ratios between the minimized reconstruction errors under regularization and the errors without regularization are 0.441 and 0.936.

For comparison, in Section S4 in Supporting Information S1, we also investigated a more traditional end‐to‐end approach to the same problem, in which we directly minimized the mean reconstruction error $∼\langle \|\hat{u}-u{\|}_{2}^{2}\rangle$ over randomly sampled spectra and noise. Although a similar noise‐robustness may eventually be obtained, we found that the popular Adam algorithm [92] for stochastic optimization converged much more slowly (exhibiting worse noise robustness if we terminated it at a number of Maxwell solves equal to our nuclear‐norm FOM with CCSA), even with hyperparameter tuning.

## 5 Concluding Remarks

Although end‐to‐end co‐design of optics and inference, incorporating training data and noise directly into the optimization process, continues to be an exciting area of research, we believe that this work illustrates new opportunities for devising inference‐related figures of merit (FOMs) for inverse design decoupled from specific inference algorithms or training data. We expect that identifying such figures of merit will be fruitful for many problems besides spectrometry, perhaps extending from polarimetry and imaging to object recognition and communications. Moreover, such FOMs, which depend only on the optical properties of the system, may be promising vehicles for identifying, proving, and approaching theoretical upper bounds on the attainable performance. Many such FOMs could potentially be explored, from linear‐algebraic quantities such as norms and condition numbers (which have many possible variations), to quantities inspired more by information theory or entropy [58, 59, 63]. We view such deterministic FOMs as a useful *complement* to end‐to‐end co‐design methods that directly minimize reconstruction error [31, 32, 33, 34, 35]: the former are more easily analyzed and may be more efficient to optimize, but the latter are more flexible in that end‐to‐end methods can be applied to more complicated reconstruction algorithms for which analytical FOMs are not yet known.

For the specific case of spectrometry, an important area of investigation is the optimal reconstruction of spectra that include both smooth background and sharp spikes (e.g., absorption or emission lines); the latter should be amenable to sparse/compressed‐sensing methods [70], but a combination of smooth and sparse methods is desirable for spectra containing both features. To optimize underdetermined sparse reconstruction, one possibility is an end‐to‐end approach [35]. Although a quantity similar to $tr{\left(FF^{⊤}\right)}^{-1}$ has been suggested as a figure of merit for underdetermined reconstruction [83], that quantity corresponds more closely to a minimum‐norm prior via the pseudoinverse [93] rather than a sparsity prior. If the spectra of interest are even more restricted, characterized by a well‐understood and extensive training set, more specialized data‐driven reconstruction strategies become applicable, such as neural networks [94]. For much larger devices operating on wider bandwidths, one could first demultiplex the bandwidth into a set of narrow windows [95, 96, 97] and then apply our methodology to spectrometry within each window using similar device footprints. Although our designs in this paper were 2D, our objective function is also applicable to 3D with the cost dominated by the Maxwell solves, which have already been demonstrated for 3D inverse designs with similar footprints [83, 84].

## Author Contributions

All authors have accepted responsibility for the entire content of this manuscript and approved its submission.

## Funding

This work was supported in part by the US Army Research Office and Air Force Research Laboratory through the Institute for Soldier Nanotechnologies (Award No. W911NF‐23‐2‐0121) and by the Simons Foundation through the Simons Collaboration on Extreme Wave Phenomena Based on Symmetries. R.P. was supported in part by the National Institutes of Health (Award No. R21EB036343). Z.L. was supported in part by the US Army Research Office (Award No. W911NF2410390) and Department of Energy (Award No. DE‐SC0024223).

## Conflicts of Interest

The authors declare no conflicts of interest.

## Supporting information


Supporting Information S1

## Data Availability

The data that support the findings of this study are available from the corresponding author upon reasonable request.
