# Supplementary material for: Inverse Design for Robust Inference in Integrated Computational Spectrometry
Source: Nanophotonics. 2026 Mar 28;15(7):e70054. doi: 10.1002/nap2.70054 (PMC13642586; doi:10.1002/nap2.70054)
Supplement: Supplementary file 1 [file NAP2-15-e70054-s001.pdf]

# Supporting Information for
# Inverse design for robust inference in integrated computational spectrometry


Wenchao Ma,[1] Raphaël Pestourie,[2] Zin Lin,[3] and Steven G. Johnson[4]

[1] *Department of Chemistry, Massachusetts Institute of Technology, Cambridge, MA 02139, USA*

[2] *School of Computational Science and Engineering, Georgia Institute of Technology, Atlanta, GA 30332, USA*

[3] *Bradley Department of Electrical and Computer Engineering, Virginia Tech, Blacksburg, VA 24060, USA*

[4] *Department of Mathematics, Massachusetts Institute of Technology, Cambridge, MA 02139, USA* *



(Dated: February 20, 2026)


**CONTENTS**


S1. Spectral reconstruction with 6 points or basis functions 1

S2. Influence of fabrication imperfections 3

S3. Analysis of objective functions 3

S4. Comparison with end-to-end design 4

S5. Integrated computational spectrometer without output waveguides 5

S6. FDTD simulation for optimization 5

References 6


## S1. SPECTRAL RECONSTRUCTION WITH 6 POINTS OR BASIS FUNCTIONS

Let us consider spectral reconstruction with 6 points or polynomials in the absence of sensor noise. As shown in Fig. S1, the results are similar to those with 7 points or basis functions shown in Fig. 5 of the main text.


* stevenj@math.mit.edu

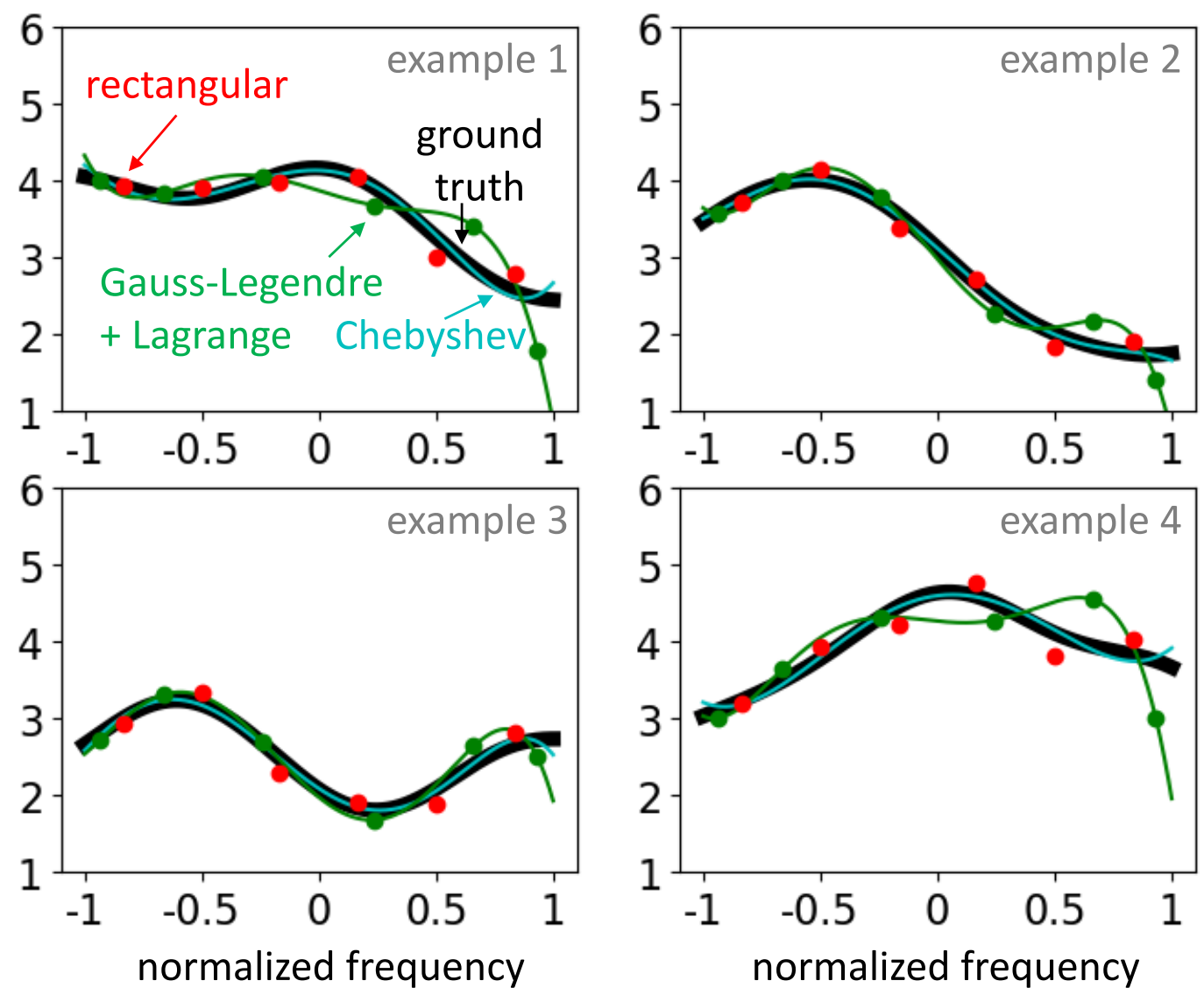


FIG. S1. Spectral reconstruction with four examples. The ground truth is plotted as thick black curves. The red dots represent reconstructed spectra at 6 equally spaced frequencies, chosen according the rectangular rule. The green dots represent reconstructed spectra at 6 frequencies chosen according to Gauss–Legendre nodes, while their Lagrange interpolating polynomials are plotted as green curves. The cyan curves represent reconstructed spectra as a linear combination of the first 6 Chebyshev polynomials of the first kind.

Apart from polynomials, radial basis functions, such as Gaussian functions, are also a typical choice. Here, we consider 6 equally spaced Gaussian functions, with an equal width and their peaks located at the same frequencies as the red dots in Fig. S1. As Fig. S2(a) shows, the relative reconstruction error, calculated in the same manner as those in Fig. 6(a) of the main text, varies with the noise level and the standard deviation $\sigma$ (proportional to the width) of the Gaussian functions. For the relative noise level $q = 0.001$, the median relative reconstruction error is minimized at $\sigma = 2.1\times$ peak spacing. At this value of $\sigma$, the variation of median relative reconstruction error with noise is illustrated as the blue curve in Fig. S2(b), with the shaded region representing the range between the first and third quartiles. For comparison, the relative reconstruction error using the first 6 Chebyshev polynomials of the first kind is also illustrated here as the magenta curve and shaded region, which is identical to those in Fig. 6(a) of the main text.

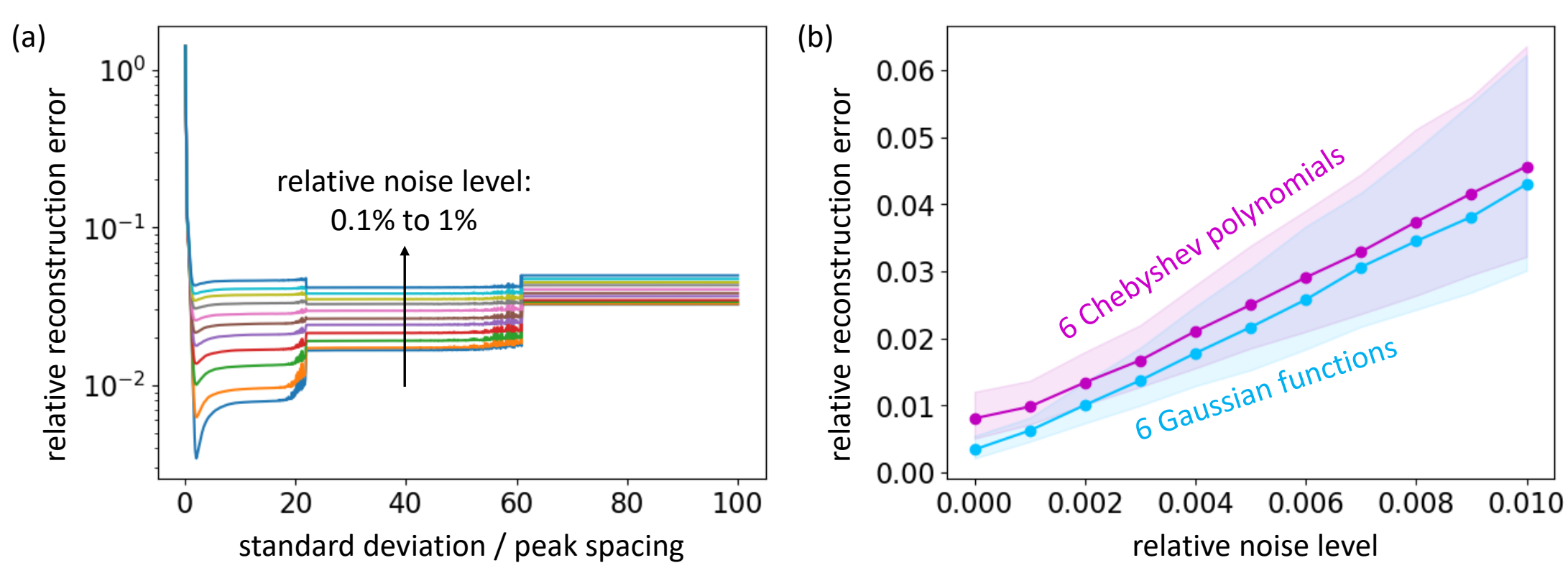


FIG. S2. Spectral reconstruction using 6 Gaussian functions, which are equally spaced and have an equal width. (a) Variation of relative reconstruction error with the standard deviation of the Gaussian functions under various relative noise levels ranging from $q = 0.001$ to 0.01. For $q = 0.001$, the median relative reconstruction error is minimized at $\sigma = 2.1\times$ peak spacing. (b) Relative reconstruction error using this value of $\sigma$. The median relative reconstruction error and the range between the first and third quartiles are represented by the blue curve and shaded region. The relative reconstruction error using the 6 Chebyshev polynomials, the same as that in Fig. 6(a) of the main text, is also illustrated for comparison, as the magenta curve and shaded region.

## S2. INFLUENCE OF FABRICATION IMPERFECTIONS

In this section, for the optimized design in Fig. 3 of the main text, we show the performance metrics under imperfections in fabrication. The influences of different extents of dilation and erosion on the optimized design pattern are illustrated in Fig. S3, while the influences of the size of the design region are shown in Fig. S4. One can see that the condition number is more vulnerable to the fabrication errors compared with transmittance. The large fabrication imperfections ($> 10$ nm) simulated here are for demonstration purposes and are not typical of realistic fabrication [1, 2].

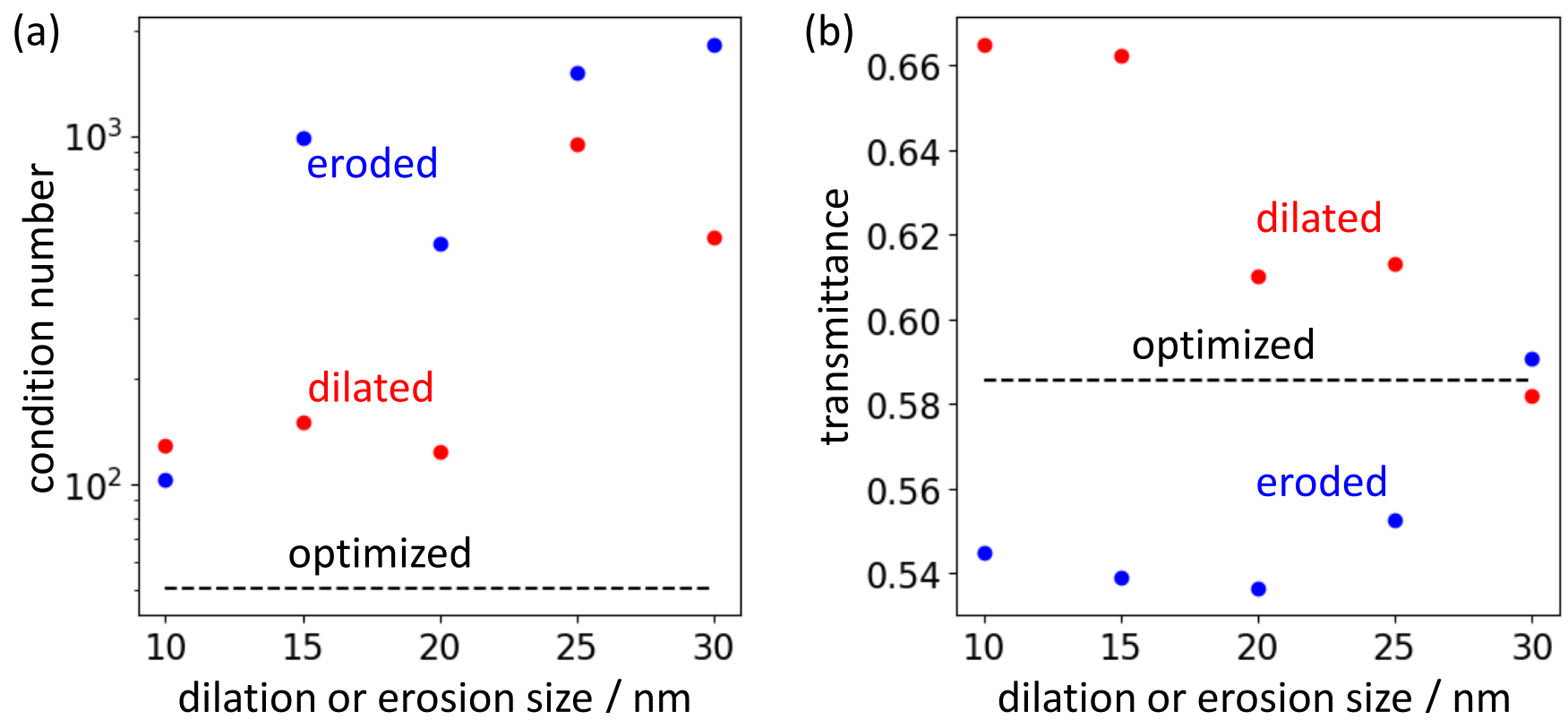


FIG. S3. Performance metrics under morphological dilation and erosion of the optimized design pattern. The red and blue dots represent the outcomes for dilated and eroded structures, respectively. The dashed lines label the performance metrics of the optimized design.

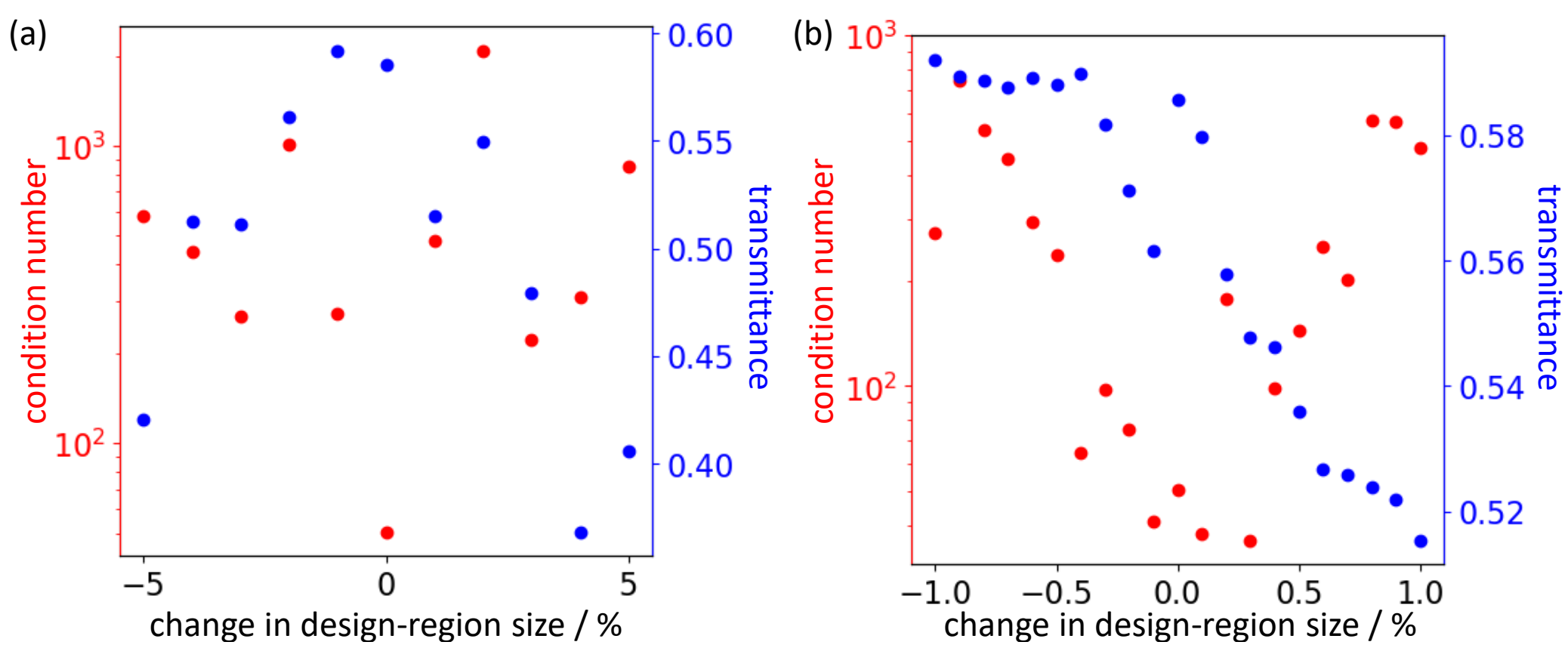


FIG. S4. Performance metrics under errors in the design-region size.

## S3. ANALYSIS OF OBJECTIVE FUNCTIONS

As introduced in the main text, a general version of our objective function to be minimized is

$$f(\rho) = \mathrm{tr}\left\{[(WF^\top F)^{-1}]^{n/2}\right\} = \sum_j {\sigma_j}^{-n}, \tag{S1}$$

where $\rho$ represents design variables; the matrix $F$ and singular values $\sigma_j$ are also functions of $\rho$ but the dependence is not written explicitly; the exponent $n$ should be positive, with $n = 1$ and $n = 2$ resulting in our objective function in Eq. (14) and the Fisher-information–inspired objective function, respectively. The function in Eq. (S1) is dominated

by the term with the smallest singular value. The gradient of the objective with respect to the design variables is

$$\nabla_\rho f = -n \sum_j \frac{\nabla_\rho \sigma_j}{{\sigma_j}^{n+1}}, \tag{S2}$$

which implies that the variation in the smallest singular value also dominates the variation of the objective. The dependence is larger for large $n$. The log-determinant objective and its gradient

$$g(\rho) = -\ln\det(WF^\top F) = -2\sum_j \ln\sigma_j, \qquad \nabla_\rho g = -2\sum_j \frac{\nabla_\rho\sigma_j}{\sigma_j}, \tag{S3}$$

also have similar dependence on the smallest singular value and its change.

## S4. COMPARISON WITH END-TO-END DESIGN

In this section, we compare the performance of our design method with end-to-end approaches and different optimization algorithms. To perform end-to-end design, a reconstruction scheme needs to be specified. Here, we consider the scheme described by Eq. (7) of the main text, which is duplicated here—with basis functions discretized as $B$ and Tikhonov regularization, the reconstructed spectrum $\hat{u}$ can be computed as

$$\hat{c} = \left[B^\top(WF^\top FW + \alpha W)B\right]^{-1}(FWB)^\top v, \qquad \hat{u} = B\hat{c}. \tag{S4}$$

In a typical end-to-end approach, the permittivity $\epsilon$ of the scatterer (which determines $F$), and possibly the regularization coefficient $\alpha$, are optimized to reduce the relative reconstruction error (backpropagating gradients "end-to-end" through both the physics and reconstruction algorithm):

$$\min_{\epsilon,\alpha}\left\langle \frac{\|\hat{u}-u\|_2^2}{\|u\|_2^2}\right\rangle, \tag{S5}$$

where the angle brackets denote averaging over spectra and noise. Ideally, an end-to-end approach would involve a denser set of frequencies to compute the measured noisy-sensor readings accurately. Here, to lower computational cost, we selected 7 frequencies according to Gauss–Legendre quadrature as an approximation. End-to-end design is typically performed with stochastic optimization, in which the average in Eq. (S5) is over the minibatch in each iteration. Here, we employed the Adam algorithm with default hyperparameters [3]. (Attempts with non-default hyperparameters did not achieve noticeably better performance in our tests.)

In Fig. S5, the relative reconstruction error from an end-to-end approach (green) with a minibatch size 1000 is compared with our nuclear-norm inverse-design approach (black), in which the trace objective is minimized with the CCSA-MMA algorithm [4]. We also minimized our nuclear-norm FOM using the Adam algorithm (red), which turned out to have slower (but more monotonic) convergence than CCSA. The noise-robustness (related to the relative reconstruction error for noisy measurements) and transmittance of the resulting design are evaluated in Fig. S5 and Table S1 for both 200 and 400 optimizer iterations (Maxwell solves). In these examples, the nuclear-norm FOM with CCSA results in better performance than either the Adam-based end-to-end approach or the Adam algorithm applied to our deterministic FOM. Although it is possible that running the Adam algorithm for long enough may eventually match the performance of the other methods, it seems to be converging more slowly—this is not too surprising, since Adam has to handle more challenging stochastic (non-deterministic) objectives. It may be that with sufficient *ad-hoc* algorithmic tuning one could improve the convergence rate of an end-to-end scheme, but we believe that greater flexibility in choosing optimization algorithms is an advantage of a determistic FOM.

TABLE S1. Transmittance of spectrometers optimized with different methods and numbers of iterations

| | nuclear-norm objective with CCSA | nuclear-norm objective with Adam | end-to-end with Adam |
|---|---|---|---|
| 200 iterations | 71.5% | 48.5% | 50.3% |
| 400 iterations | 76.4% | 53.7% | 56.3% |

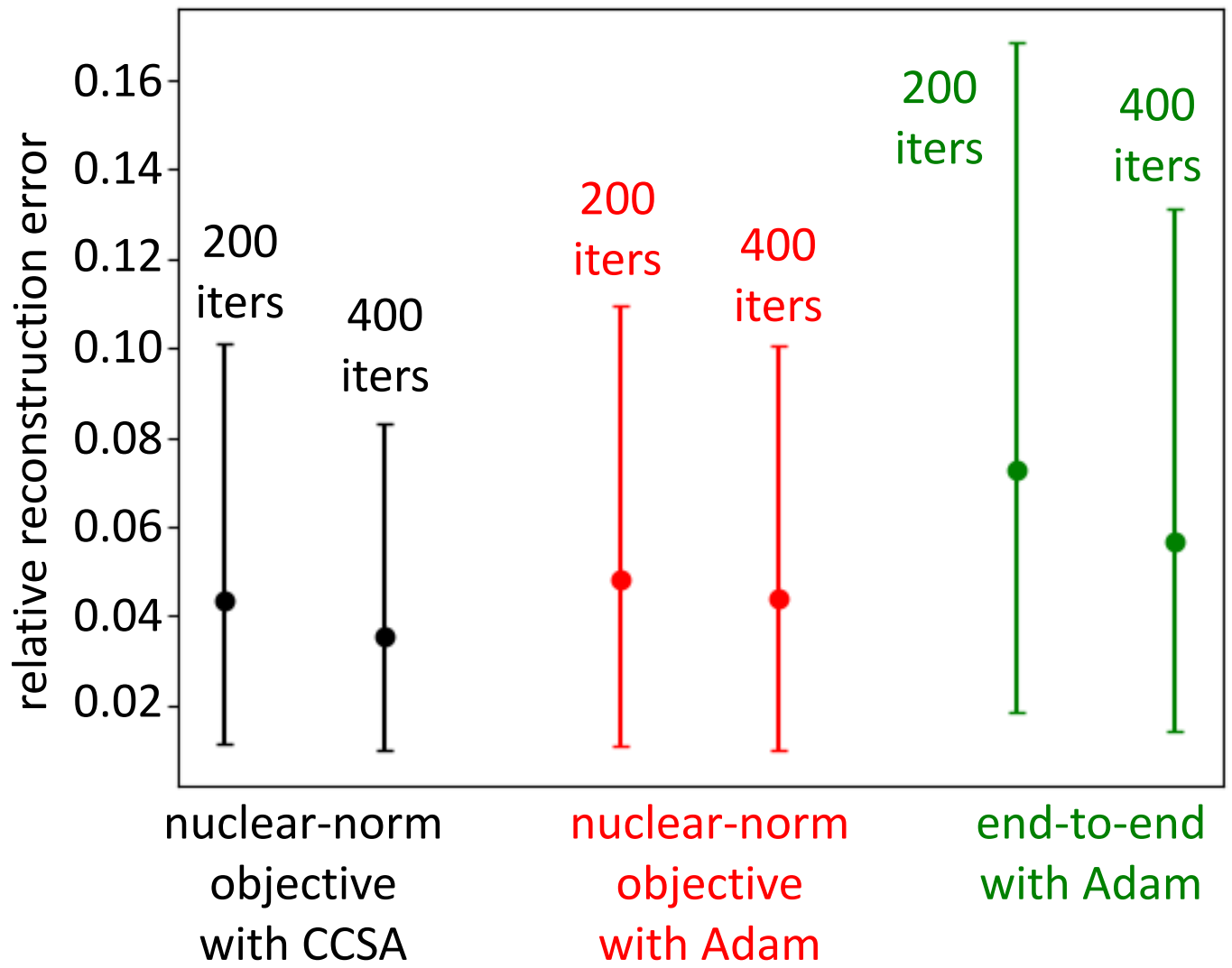


FIG. S5. Reconstruction errors associated with spectrometers designed by different methods. The relative reconstruction error denoted by the vertical axis is computed as $\sqrt{\frac{1}{\omega_{\max}-\omega_{\min}}\int[\hat{u}(\omega)-u(\omega)]^2d\omega}/\langle u(\omega)\rangle$ with $\langle u(\omega)\rangle = u_{\text{avg}}$, which is the same as that in Fig. 6 of the main text. The integral is evaluated at 101 frequencies located at the (scaled) Gauss–Legendre quadrature nodes. Each dot represents the median among $10^5$ cases with randomly generated spectra and noise according to Eqs. (16) and (17) in the main text. Each error bar ranges from the first to third quartiles. The examples here are based on the same integrated structure as that sketched in Fig. 3(a) of the main text, but with the hyperparameter $\beta$ fixed at 8. The regularization coefficient $\alpha$ was optimized (or reoptimized if the end-to-end approach is used) after the design was given.

## S5. INTEGRATED COMPUTATIONAL SPECTROMETER WITHOUT OUTPUT WAVEGUIDES

Here, we demonstrate our methods on a simple two-dimensional (2d, $xy$) example of an integrated spectrometer, in which output signals are collected by sensors on a screen distant from the scatterer, as Fig. S6(a) shows. The input waveguide, the wedge region, and the design region have the same sizes and relative positions as those in Fig. 3(a) of the main text. The output waveguides are replaced by free space, while 12 sensors, each with a size of 2 µm, are closely arranged on a screen parallel to the design region with a distance of 12 µm, as depicted in Fig. S6(a). The signal recorded by each sensor is proportional to the power of waves traveling through that sensor. Incoming waves have wavelengths between 1.54 and 1.56 µm with out-of-plane ($E_z$) polarization, which are the same as those in the main text. We selected seven frequencies according to Gauss–Legendre quadrature of $\int_{1.54}^{1.56} d\lambda$ for performing inverse design, so the size of the spectral–spatial mapping matrix is again $12 \times 7$.

As shown in Fig. S6, the process and result of inverse design are similar to those in Fig. 3(a) of the main text. However, in comparison with random structures with the same minimum lengthscale (80 nm), the optimized design has moderate total transmittance but much lower condition number, as Fig. S7 illustrates.

## S6. FDTD SIMULATION FOR OPTIMIZATION

All electromagnetic simulations in optimization and verification were performed with a free and open-source implementation of the finite-difference time-domain (FDTD) method [5]. The inverse design and end-to-end design were performed with the hybrid time/frequency-domain adjoint module of Meep. In the design process, the size of the simulation cell is 14 µm × 6 µm, whose periphery is a perfectly matched layer (PML) with a thickness of 1 µm. The resolution of FDTD simulation is 50 pixels/µm, while the resolution of the material grid, which is separate from the simulation resolution, is 200 pixels/µm. Seven frequencies are involved.

Each iteration in optimization is composed of a forward simulation and an adjoint simulation. On a supercomputing node with 48 CPUs (Intel Xeon Platinum 8260), an iteration in designing the spectrometer in the main text takes 200 to 300 sec, while an iteration in designing the spectrometer in Sec. S5 takes 700 to 800 sec. The design processes

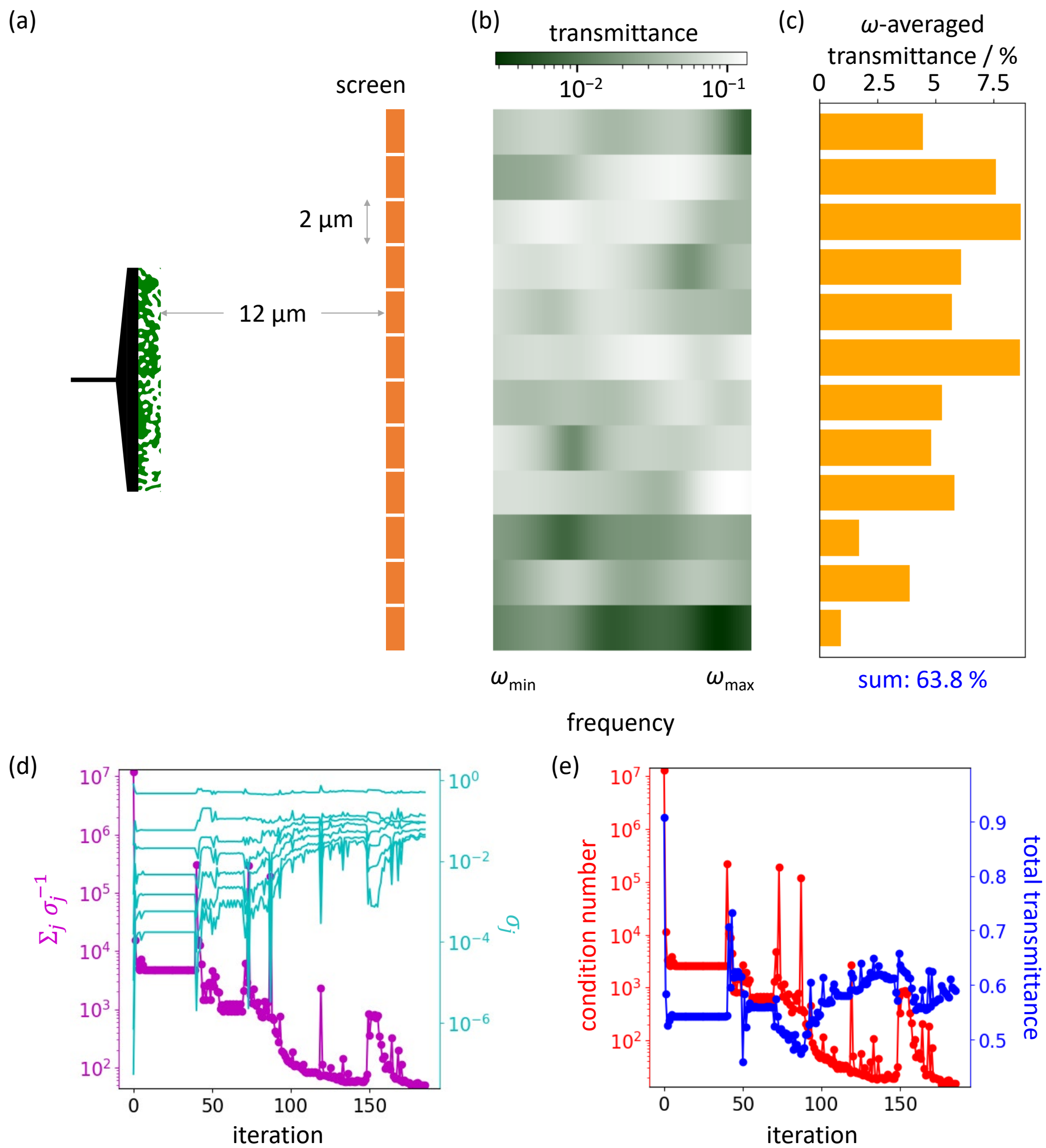


FIG. S6. Inverse design of an integrated computational spectrometer without output waveguides. (a) Structure of the spectrometer. This 2d structure consists of an input waveguide, a wedge region, a design region, and a screen equipped with 12 sensors, with the solid material having a relative permittivity ≈ 12. The sizes of each sensor and the screen are 2 μm and 24 μm, respectively. Apart from the free space and screen in place of the output waveguides, the other components are the same as those in Fig. 3(a) of the main text. (b) Transmittance of the optimized spectrometer at each output waveguide across the frequency range of interest. (c) Frequency-averaged transmittance of the optimized spectrometer at each output waveguide. The total transmittance is 63.8%. (d) Objective function ($\sum_j \sigma_j^{-1}$) and singular values during optimization. (e) Condition number and total transmittance during optimization, computed from the $12 \times 7$ spectral–spatial mapping matrix.

in the two cases undergo roughly 300 or 200 iterations, which take about one or two days in total.

---

[1] S. Y. Siew, B. Li, F. Gao, H. Y. Zheng, W. Zhang, P. Guo, S. W. Xie, A. Song, B. Dong, L. W. Luo, C. Li, X. Luo, and G.-Q. Lo, Review of silicon photonics technology and platform development, Journal of Lightwave Technology **39**, 4374–4389 (2021).

[2] D. S. Boning, S. I. El-Henawy, and Z. Zhang, Variation-aware methods and models for silicon photonic design-for-manufacturability, Journal of Lightwave Technology **40**, 1776–1783 (2022).

[3] D. P. Kingma and J. Ba, Adam: A method for stochastic optimization (2014).

[4] K. Svanberg, A class of globally convergent optimization methods based on conservative convex separable approximations, SIAM Journal on Optimization **12**, 555–573 (2002).

[5] A. F. Oskooi, D. Roundy, M. Ibanescu, P. Bermel, J. Joannopoulos, and S. G. Johnson, Meep: A flexible free-software package for electromagnetic simulations by the fdtd method, Computer Physics Communications **181**, 687–702 (2010).

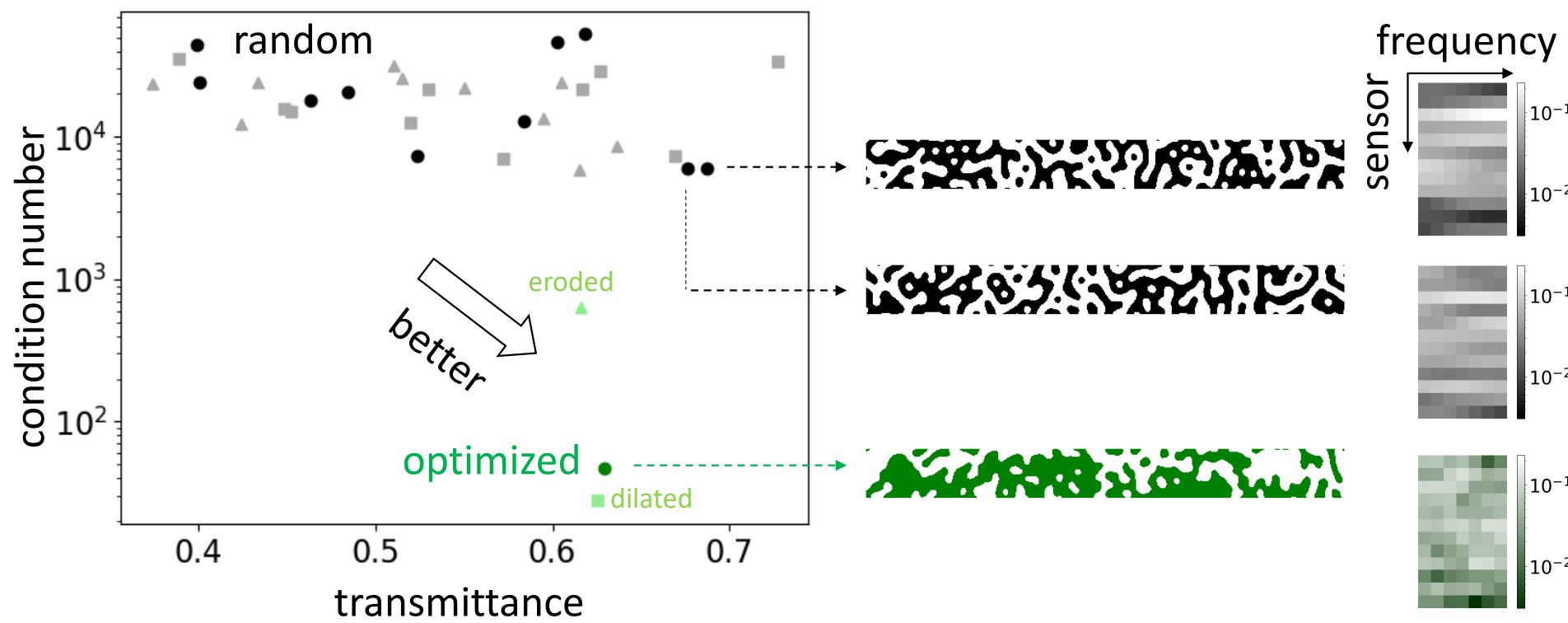


FIG. S7. Comparison of performances of random and optimized structures. The horizontal and vertical axes represent the total transmittance and the condition number, respectively. The bottom right area is associated with lower condition numbers, higher total transmittance, and hence better performance. The round green and black dots correspond to the optimized and random structures with the same minimum lengthscale, while the square/triangular symbols with light colors correspond to structures in which solid regions are dilated/eroded by 10 nm. The optimized structure and two random structures are illustrated on the middle panel. Their corresponding $12 \times 7$ spectral–spatial mapping matrices are on the right panel.
